# Task-dependent inhibition of slow-twitch soleus and excitation of fast-twitch gastrocnemius do not require high movement speed and velocity-dependent sensory feedback

**DOI:** 10.3389/fphys.2014.00410

**Published:** 2014-10-28

**Authors:** Ricky Mehta, Boris I. Prilutsky

**Affiliations:** Center for Human Movement Studies, School of Applied Physiology, Georgia Institute of TechnologyAtlanta, GA, USA

**Keywords:** triceps surae, inhibition of soleus, muscle coordination, electromyography, paw shake, walking, load lifting, jumping

## Abstract

Although individual heads of triceps surae, soleus (SO) and medial gastrocnemius (MG) muscles, are often considered close functional synergists, previous studies have shown distinct activity patterns between them in some motor behaviors. The goal of this study was to test two hypotheses explaining inhibition of slow SO with respect to fast MG: (1) inhibition occurs at high movement velocities and mediated by velocity-dependent sensory feedback and (2) inhibition depends on the ankle-knee joint moment combination and does not require high movement velocities. The hypotheses were tested by comparing the SO EMG/MG EMG ratio during fast and slow motor behaviors (cat paw shake responses vs. back, straight leg load lifting in humans), which had the same ankle extension-knee flexion moment combination; and during fast and slow behaviors with the ankle extension-knee extension moment combination (human vertical jumping and stance phase of walking in cats and leg load lifting in humans). In addition, SO EMG/MG EMG ratio was determined during cat paw shake responses and walking before and after removal of stretch velocity-dependent sensory feedback by self-reinnervating SO and/or gastrocnemius. We found the ratio SO EMG/MG EMG below 1 (*p* < 0.05) during fast paw shake responses and slow back load lifting, requiring the ankle extension-knee flexion moment combination; whereas the ratio SO EMG/MG EMG was above 1 (*p* < 0.05) during fast vertical jumping and slow tasks of walking and leg load lifting, requiring ankle extension-knee extension moments. Removal of velocity-dependent sensory feedback did not affect the SO EMG/MG EMG ratio in cats. We concluded that the relative inhibition of SO does not require high muscle velocities, depends on ankle-knee moment combinations, and is mechanically advantageous for allowing a greater MG contribution to ankle extension and knee flexion moments.

## Introduction

Skeletal muscles have diverse morphological properties and can differ substantially in muscle fiber type composition, fascicle length, physiological cross-sectional area, pennation, tendon length and thickness, etc., (Ariano et al., 1973; Johnson et al., 1973; Sacks and Roy, 1982; Wood et al., 1989; Cutts et al., 1991; Ward et al., 2009). Distinct morphological properties of muscle fascicle groups within a muscle, i.e., muscle compartments, are also well documented. Single muscle compartments are separated by a sheath of connective tissue and often differ in muscle fiber architecture, fiber histochemical properties, mechanical action at the joint and innervation (English and Letbetter, 1982; Loeb et al., 1987; Segal et al., 1991; Nichols, 1994; Richmond et al., 1999). As a result, activity patterns and mechanical actions of individual muscles within a synergistic muscle group or those of individual muscle compartments within a muscle are distinct and task-dependent (English, 1984; Chanaud and Macpherson, 1991; Lawrence et al., 1993; Carrasco and English, 1999; Brown et al., 2007). The diversity of morphological properties and mechanical actions of individual muscles and muscle compartments has been explained by a wide range of functional demands on the animal during everyday motor behaviors (Loeb, 1985; Otten, 1988; Gans and Gaunt, 1991; Benjamin et al., 2008).

Some large skeletal muscles, e.g., triceps surae, quadriceps, hamstrings and triceps brachii, consist of individual muscle heads that not only differ from each other in their morphological properties, but also in the number of joints they cross. For example, one-joint ankle extensor soleus (SO) and two-joint ankle extensor and knee flexor gastrocnemius (GA) are individual muscle heads of triceps surae. The fact that GA can contribute to both ankle extension and knee flexion moments gives this muscle head a mechanical advantage over SO in tasks that require this combination of joint moments; this is also true for two-joint heads of the quadriceps, hamstrings and triceps brachii muscles mentioned above (Wells and Evans, 1987; Prilutsky, 2000a).

Although GA and SO, being separate muscle heads of triceps surae, are often considered close anatomical and functional synergists and show similar activity patterns in many motor tasks [e.g., during level walking, running (Prilutsky and Gregor, 2001; Cappellini et al., 2006; Markin et al., 2012) and jumping (Bobbert and Van Ingen Schenau, 1988; Pandy and Zajac, 1991; Kurokawa et al., 2001)], studies have also shown distinct activity or force patterns between SO and GA across different speeds or slopes of walking and running (Walmsley et al., 1978; Prilutsky et al., 1996; Kaya et al., 2003; Neptune and Sasaki, 2005), cycling (Ryan and Gregor, 1992), cat paw-shake response (Smith et al., 1980; Abraham and Loeb, 1985; Fowler et al., 1988), and external force control in humans (Wells and Evans, 1987; Jacobs and Van Ingen Schenau, 1992). The reasons and mechanisms explaining differential activity of the individual one-joint and two-joint heads of the large limb muscles, and specifically triceps surae, in some tasks but not others are still debated.

Several researchers have suggested that the differential inhibition of the slow-twitch SO but not the fast-twitch GA during fast movements such as cat paw shaking (Smith et al., 1980), cat upslope walking (Kaya et al., 2003) and human fast walking (Neptune and Sasaki, 2005) is advantageous because the contracting SO might not be able to contribute much force to and slow down the ongoing fast movement. It seems reasonable to suggest that the differential inhibition of slow-twitch SO and excitation of fast-twitch GA muscles during fast movements could be mediated via the velocity-sensitive spindle Ia afferents (e.g., Smith and Zernicke, 1987; Labella et al., 1989). For example, during fast cat paw shake responses accompanied by inhibition of SO and high EMG activity of GA, extremely high firing rates of the primary spindle afferents from triceps surae have been reported to occur in phase with stretches and EMG bursts of triceps surae (Prochazka et al., 1977, 1989). On the other hand, removal of proprioceptive inputs from muscle afferents by cutting the muscle nerve does not affect the recruitment order of motor units in the cat mixed medial gastrocnemius (MG) muscle from slow-twitch to fast-twitch during evoked stretch and cutaneous reflexes in the decerebrate cat (Haftel et al., 2001). Thus, the neural mechanisms by which slow-twitch motor units during fast movements are inhibited are still unclear.

Another explanation of the functional significance of the differential activation of SO and GA seen in some motor behaviors might not be directly related to the difference in muscle fiber type composition of these triceps surae heads, but potentially depend on the number of joints they cross. It has been shown that during tasks in which GA, a two-joint muscle, has agonistic actions at both joints it crosses, i.e., contributes to resultant ankle extension and knee flexion moments, its EMG activity is much higher than at other combinations of ankle and knee joint moments. This GA behavior has been documented in back (straight-leg) load lifting (Prilutsky et al., 1998; Mehta and Prilutsky, 2012), cycling (Prilutsky and Gregor, 2000) and exertion of leg force on the external environment in humans (Wells and Evans, 1987; Jacobs and Van Ingen Schenau, 1992). This activation strategy of GA, and other two-joint muscles, has been shown to be mechanically advantageous: it allows for minimization of the total muscle stress and fatigue in musculoskeletal models of the human leg performing variety of motor tasks (Prilutsky and Gregor, 1997; Prilutsky et al., 1998; Prilutsky, 2000a; Raikova and Prilutsky, 2001; Prilutsky and Zatsiorsky, 2002). The total muscle force requirement for producing the ankle extension and knee flexion joint moments would be substantially reduced if these moments are primarily produced by two-joint ankle extensors and knee flexors, such as GA, compared to one-joint ankle extensors (SO) and knee flexors (short head of biceps femoris). A neural mechanism that might be responsible for such behavior has been suggested to originate from two independent inputs to motoneuronal pools of two-joint muscles from spinal interneuron circuitry comprising flexor and extensor half-centers of spinal pattern generator networks (Perret and Cabelguen, 1980; Prilutsky, 2000a; Shevtsova et al., 2014). A neural mechanism to satisfy the required moments at adjacent joints while ensuring a greater contribution of the two-joint muscles spanning these joints has been postulated—a force-dependent inhibition from a two-joint muscle to its one-joint synergists (Prilutsky, 2000a). Such force-dependent inhibition from GA to SO and from rectus femoris (knee extensor and hip flexor) to vastii (one-joint knee extensors) has in fact been reported for muscle stretch evoked responses in the decelebrate cat (Nichols, 1994, 1999; Wilmink and Nichols, 2003).

The goal of this study was to test the two possible explanations emerged from the literature for the differential activation of SO and GA—(1) SO inhibition occurs at high movement velocities and mediated by velocity-dependent sensory feedback and (2) SO inhibition depends on the ankle-knee joint moment combination and does not require high movement velocities. The hypotheses were tested by comparing the SO EMG/MG EMG ratio during fast and slow motor behaviors—cat paw shake responses vs. back, straight leg load lifting in humans, which had the same ankle extension-knee flexion moment combination (De Looze et al., 1993; Prilutsky et al., 1998, 2004; Klishko et al., 2011); and during fast and slow behaviors with the ankle extension-knee extension moment combination—human vertical jumping (Bobbert and Van Ingen Schenau, 1988; Kurokawa et al., 2001), stance phase of walking in cats (Gregor et al., 2006) and leg load lifting in humans (De Looze et al., 1993).

If the ratio SO EMG/MG EMG was found to be lower in fast tasks (paw shaking and jumping) than in slow tasks (load lifting and walking) irrespective of joint moment combinations, hypothesis 1 would be supported and hypothesis 2 rejected. To test if SO inhibition is mediated by velocity-sensitive sensory feedback from SO and GA, EMG activity of SO and MG was recorded during cat paw-shake responses and level walking in the cat before and after velocity-sensitive Ia afferent feedback from SO and lateral gastrocnemius (LG) or MG and LG was removed by self-reinnervation of these muscles (Cope and Clark, 1993; Cope et al., 1994). After self-reinnervation of ankle extensors (transecting and repairing the nerves innervating the muscles), locomotor muscle activity recovers in several months (O'Donovan et al., 1985; Gregor et al., 2003), however self-reinnervated muscles permanently loose stretch-reflex (Cope and Clark, 1993; Cope et al., 1994) due to retraction of excitatory synapses with motoneurons by primary spindle afferents (Alvarez et al., 2011; Bullinger et al., 2011). If SO inhibition, judged by the ratio SO EMG/MG EMG, during paw shake responses was reduced (the ratio increased) after SO and/or GA self-reinnervation, the hypothesis that SO inhibition requires high movement velocity and mediated by velocity-sensitive afferent feedback from primary spindle afferents would be supported.

Preliminary results have been published in abstract form (Prilutsky et al., 2004; Klishko et al., 2011; Mehta and Prilutsky, 2012).

## Material and methods

### Cat experiments

#### Animals and surgical procedures

The surgical and experimental procedures employed in the study corresponded to the “Principles of Laboratory Animal Care” (NIH Publication No. 86–23, Revised 1985) and were approved by the Institutional Animal Care and Use Committee of the Georgia Institute of Technology. Six female, adult cats (*felis domesticus*, mass = 3.5 ± 0.8 kg) were used in this study. Prior to any surgery, cats were trained using food reward to walk on a Plexiglas enclosed walkway (3.0 × 0.4 m^2^) with up to three embedded force plates (16 × 11 and 11 × 7 cm^2^; Bertec Corporation, Columbus, OH, USA) for several hours a day, 5 days a week for 3–4 weeks; for more details see Prilutsky et al. (2005); Gregor et al. (2006). A brief description of surgical procedures will be given since they have been described in detail previously (Gregor et al., 2006; Prilutsky et al., 2011; Hodson-Tole et al., 2012). Two survival surgeries were performed under asceptic conditions using general isoflurane anaesthesia.

***Implantation surgery***. Anaesthesia was induced by ketamine (10 mg/kg, s.c.), atropine (0.05 mg/kg, s.c.) and isoflurane (inhalation, 5%) and maintained with isoflurane (1–3%). Vital physiological characteristics (temperature, respiration, heart rate and blood pressure) were monitored throughout the surgery. Teflon-insulated multi-stranded stainless steel fine wires (CW5402; Cooner Wire, Chatsworth, CA, USA) attached to a multi-pin Amphenol connector were passed subcutaneously from skin incision on the scull along the back toward the right hindlimb. The connector was attached to the skull using four stainless steel or titanium screws and dental cement. A small strip of insulation (~1 mm) was removed near the distal end of each wire and a pair of wires was implanted in the mid belly of MG and SO muscles of the right hindlimb (the distance between wires in each muscle was approximately 5–10 mm). Only one medial head of GA muscle was tested in this study since the two MG and LG heads of triceps surae have similar morphological properties, mechanical actions at the ankle joint and activity patterns during walking and paw shake response (Ariano et al., 1973; Sacks and Roy, 1982; Abraham and Loeb, 1985; Lawrence et al., 1993; Gregor et al., 2006). Skin incisions were closed using Vicryl 4–0 suture for the deep fascia and Vicryl 5–0 suture for subcuticular closure. The animal recovered after surgery for 14 days with pain medication (fentanyl transdermal patch, 12–25 μg/h and/or buprenorphine, s.c., 0.01 mg/kg, or ketoprofen, 2 mg/kg, s.c.) administered for at least 3 days and antibiotics (cefovecin, 8 mg/kg, s.c., or ceftiofur, 4 mg/kg, s.c.) for 10 days.

***Nerve transection and repair surgery***. Details of nerve transection and repair have been described previously (Maas et al., 2007, 2010; Prilutsky et al., 2011). Anesthetics during the surgery and pain medication following the surgery were the same as those for the implantation surgery. A longitudinal incision in the popliteal region of the right hindlimb was made in order to identify and isolate the branches of the tibial nerve which innervate single heads of triceps surae—SO, LG, and MG muscles. After dissecting surrounding tissue and leaving the fat pad intact, the MG and LG nerves in 2 cats and the LG-SO nerve in 4 cats were transected using sharp scissors, carefully aligned together and sutured using 10–0 non-absorbable nylon or fixed in place using fibrin glue [equal parts of thrombin and a 1:1 mixture of fibrin and fibronectin; Sigma-Aldrich, St. Louis, MO, USA (English, 2005)], respectively. Before experiments were resumed, the animal recovered for 3–5 days following the surgery (Prilutsky et al., 2011).

#### Data collection

Animal kinematics and electromyographic (EMG) activity were recorded during upslope (27°) and level walking and paw shake responses before and at least 12 weeks after nerve transection and repair. The period of 12 weeks was sufficient for the injured muscles to be reinnervated and regain the pre nerve transection EMG activity level (O'Donovan et al., 1985; Gregor et al., 2003; Pantall et al., 2012).

To record kinematics of walking and paw shake responses, light reflective markers were attached by double-sided adhesive tape to shaved skin overlying the following right hindlimb bony landmarks: the iliac crest, greater trochanter, lateral femoral epicondyle, lateral malleolus, 5th metatarsophalangeal (MTP) joint and the distal end of the 5th digit. Marker displacements were recorded using a 3D, 6-camera motion capture system (Vicon Motion Systems Ltd, Oxford, UK) at a sampling rate of 120 Hz. Ground reaction forces were collected during walking using embedded force plates (Bertec Corporation, Columbus, OH, USA) at 360 Hz. A 16-conductor, shielded flexible cable was connected to the Amphenol connector on the cat's head to record EMG activity. EMG and mechanics data collection was synchronized by an electronic trigger pulse from the Vicon system. EMG signals were recorded at 3000 Hz, band-pass filtered (30–1000 Hz, 3 dB), amplified (100×) and saved on a computer for further analysis.

Two motor behaviors were studied—level walking, a slow task, in which ankle extension and knee extension moments are developed during the stance phase (Gregor et al., 2006; Prilutsky et al., 2011); and paw shake responses, a fast task, in which ankle extension and knee flexion moments are produced simultaneously during one half of the paw shake cycle (Hoy et al., 1985; Prilutsky et al., 2004). Paw shake responses (PSRs) were elicited by placement of a small piece of adhesive tape on paw pad of the right hindlimb. The animal was then placed in the recording area and was free to walk along a Plexiglas enclosed walkway. This allowed for the collection of several unconstrained PSRs which occurred during swing phases of walking while the cat stopped and shook the hindlimb before continuing walking. In some cases PSRs occurred spontaneously during recordings of level walking. PSR and locomotion experiments were performed both before and at least 12 weeks after LG-MG and SO-LG nerve transection and repair.

#### Cat data analysis and statistics

**Figure 1:**
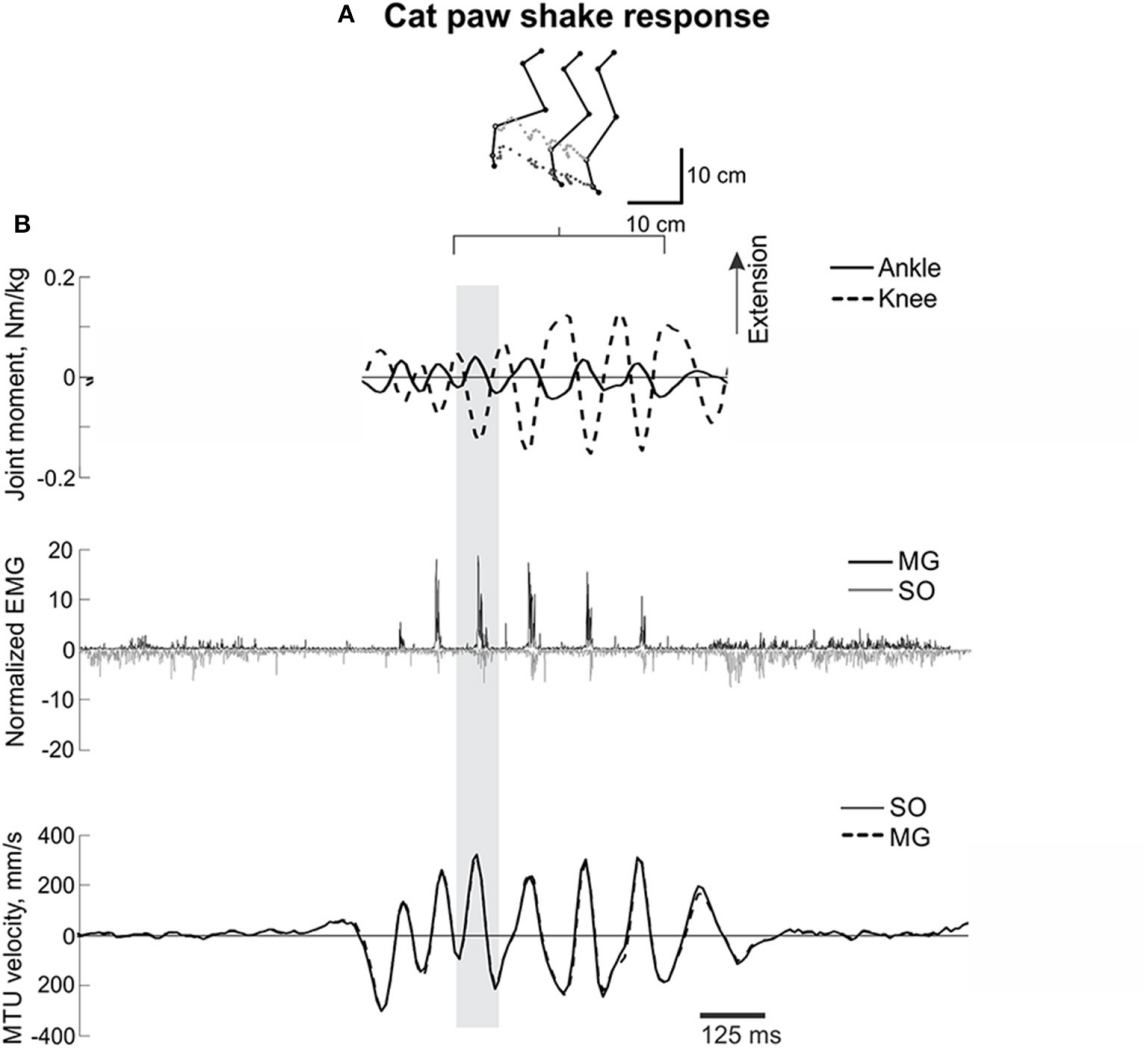
**A representative episode of a cat paw shake response**. **(A)** Stick figures and trajectories of the right metatarsophalangeal and ankle joints during several cycles of paw shake. **(B)** Normalized resultant muscle moments at the ankle and knee (top, positive values designate extension); normalized raw, full-wave rectified EMG activity (middle traces) of MG (positive values) and SO (negative values); and MTU velocity (bottom traces) of MG and SO, where positive MTU velocity corresponds to lengthening. The gray rectangle indicates a single paw shake cycle.

**Figure 2:**
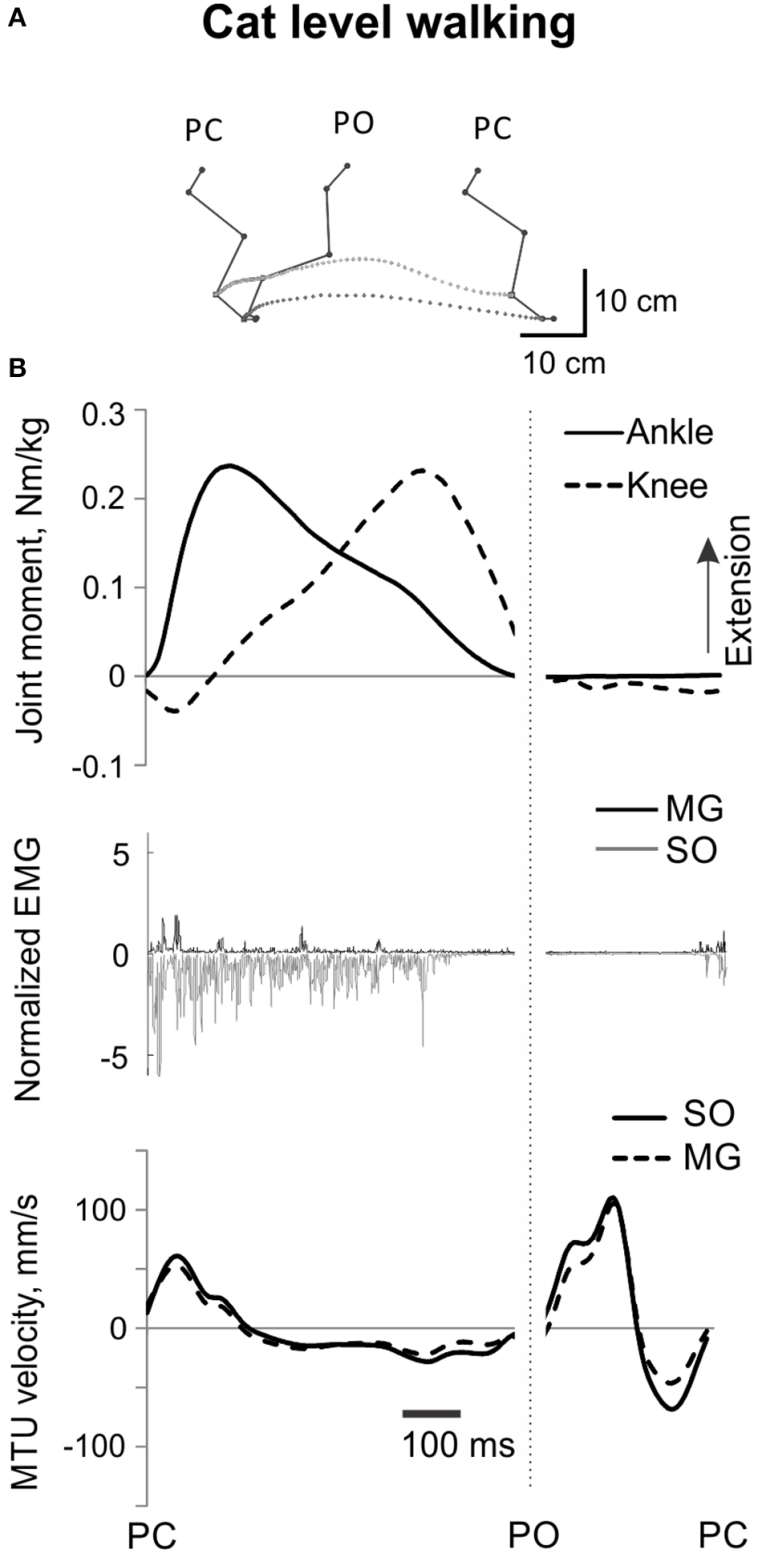
**A representative cycle of cat level walking**. **(A)** Stick figures and trajectories of the right metatarsophalangeal and ankle joints during a walking cycle. **(B)** Normalized resultant muscle moments at the ankle and knee (top, positive values designate extension); normalized raw, full-wave rectified EMG activity (middle traces) of MG (positive values) and SO (negative values); and MTU velocity (bottom traces) of MG and SO, positive MTU velocity corresponds to lengthening. Dotted vertical line separates stance and swing phase; PC, paw contact; PO, paw off.

EMG activity and hindlimb mechanics during PSR episodes (Figure 1) and walking cycles (Figure 2) were analyzed using custom software (Prilutsky et al., 2005; Gregor et al., 2006). Walking and PSR cycles were identified using vertical ground reaction forces produced by the right hindlimb or recorded kinematics, respectively. Joint angles were calculated after filtering recorded marker coordinates using a fourth order, zero lag Butterworth filter (10 and 15 Hz cut-off frequencies for walking and paw shakes, respectively; and muscle-tendon unit (MTU) lengths of SO and MG were calculated using a scaled geometric model of the cat hindlimb assuming a straight muscle path between muscle origin and insertion (Goslow et al., 1973; Gregor et al., 2006). Muscle velocity was calculated as the time-derivative of MTU length using the method of finite differences. The resultant muscle moments at the hindlimb joints in the sagittal plane were computed using a standard inverse dynamics analysis described in detail elsewhere (Prilutsky et al., 2005, 2011; Gregor et al., 2006). Inertial properties of cat hindlimb segments necessary for calculations were obtained using the regression equations from Hoy and Zernicke (1985). The computed moments were normalized by cat mass.

Recorded and band-passed EMG signals (see above) were full-wave rectified and EMG burst onset and offset times determined either by observing the signal on computer screen or by a computer program using a 2-SD threshold above the EMG baseline observed during the swing phase of walking (Gregor et al., 2006). The mean burst EMG activity was computed for each cycle of level walking or paw shake response. Full-wave rectified signals were also low-pass filtered (fourth order, zero lag Butterworth filter, cut-of frequency of 30 and 75 Hz for walking and paw shake, respectively) to obtain EMG linear envelopes. The mean EMG burst and linear EMG envelope values were normalized to the maximum mean burst EMG magnitude found across all walking conditions (level and upslope) before nerve cut and repair for each cat and muscle.

For further PSR analysis, individual steady-state cycles in the middle of paw shake episodes were visually identified using consecutive peaks of the ankle flexion moment considered as the start and end of the paw shake cycle (gray rectangle in Figure 1). In total, 124 individual PSR cycles were identified across all cats and PSR episodes. For each PSR cycle, the mean ankle extension and knee flexion moments were calculated during the phase of the cycle in which this combination of joint moments and EMG bursts of SO and MG occurred (Figure 1). The SO and MG EMG bursts identified as described above were also averaged using the full-wave rectified signals and then normalized by the corresponding maximum mean EMG values for SO and MG muscles found across all recorded walking cycles for a given cat before nerve cut and repair.

All analyzed mechanical and EMG variables obtained for 45 cycles of level walking across all cats before and after self-reinnervation were time-normalized to the duration of the stance and swing phase separately. The mean ankle and knee joint moments and mean SO and MG EMG activity were calculated for the stance phase of walking (from paw contact, PC to paw-off, PO), during which the ankle and knee joint moments were mostly extension and most of SO and MG EMG bursts occurred (Figure 2). The mean SO and MG EMG values were normalized to the maximum mean EMG burst of each muscle as described above. For each stance phase of level walking and each PSR cycle, the ratio (mean normalized SO EMG / mean normalized MG EMG) was calculated.

All statistical tests were performed using IBM SPSS Statistics v20 software (IBM SPSS, Chicago, IL, USA). A linear mixed model analysis was used to test several hypotheses: (1) the ratio SO EMG/MG EMG would be smaller (or SO inhibition would be greater) at ankle extension-knee flexion combinations of joint moments (during fast PSRs) than at ankle extension-knee extension combinations of joint moments (during a slow task of walking, Table 1); and (2) removal of velocity-dependent feedback from SO and/or GA muscles (by self-reinnervation) would increase the ratio SO EMG/MG EMG during PSRs (would reduce SO inhibition with respect to GA).

**Table 1:** **Subjects and experimental tasks with different velocity demands and combinations of ankle and knee joint moments**.

	**Movement speed**	**Slow**	**Fast**
**Joint moment combination**			
Ankle extension–knee extension		Stance of walking (pre/post-reinnervation: 5/4 cats, 20/25 cycles, *V*_MG_ = 0.115 ± 0.039, *V*_SO_ = 0.203 ± 0.067) Leg load lifting (5% BW) (5 humans, 29 trials, *V*_MG_ = 0.020 ± 0.003, *V*_SO_ = 0.081 ± 0.031)	Vertical jump (5 humans, 40 trials, *V*_MG_ = 0.241 ± 0.034, *V*_SO_ = 1.559 ± 0.136)
Ankle extension–knee flexion		Back load lifting (5/15% BW: 5/5 humans, 25/28 trials, *V*_MG_ = 0.020 ± 0.009, *V*_SO_ = 0.063 ± 0.021)	Paw shake response (pre/post-reinnervation: 5/4 cats, 62/62 cycles, *V*_MG_ = 0.473 ± 0.353, *V*_SO_ = 0.814 ± 0.482)

*BW is body weight, *V*_MG_ and *V*_SO_ are the peaks of MTU shortening velocities of MG and SO, respectively, normalized to the maximum shortening velocity of the corresponding muscle. Maximum shortening velocities of cat SO = 176 mm/s and MG = 259 mm/s were taken from measurements of Spector et al. (1980); maximum shortening velocities of human SO = 172 and MG = 683 mm/s were taken from estimates of Prilutsky (2000b). Although the peak of human SO MTU shortening velocity during jumping exceeds its SO maximum velocity, SO fascicles shorten at a much lower velocity during jumping (Kurokawa et al., 2001)*.

In the linear mixed model analysis, the dependent variables were the SO EMG/MG EMG ratio and the mean normalized SO and MG EMG burst magnitude. The fixed factors were the joint moment combination (ankle extension-knee flexion, in PSR, and ankle extension-knee extension, in stance of walking) and muscle proprioception status (pre and post self-reinnervation). Individual cats and PSR or walking cycles were considered random factors. The Bonferroni *post-hoc* test was used for pairwise comparisons. One-sample *t*-tests were also performed to determine whether the SO EMG/MG EMG ratio for each task was significantly different from a test value of 1; the latter value indicates an equal normalized activity of SO and MG. Descriptive statistics values are reported as mean ± *SD*. Significance level for all tests was set at an alpha level of 0.05.

### Human experiments

#### Participants

Five healthy adults (4 males, 1 female; age = 31 ± 12 years, mass = 78 ± 9.0 kg, height = 1.7 ± 0.1 m) participated in this study. All participants reviewed and signed an informed consent form approved by the Institutional Review Board of the Georgia Institute of Technology prior to the study. Participants were recruited if they were over the age of 18 and had no history of known musculoskeletal or neurological disorders.

#### Participant preparation

Participants were first prepared for placing EMG surface electrodes (Noraxon Inc., Scottsdale, AZ, USA; 1 cm diameter, 2 cm inter-electrode distance), which included shaving and lightly rubbing the skin with an alcohol pad. Locations of the muscles were found by palpation of the triceps surae while the participant was performing active contractions. After finding appropriate locations for electrode placement in the mid-belly of SO and MG muscles, the electrodes with the pre-amplifiers and cables were secured in place with adhesive tape and elastic bandage to reduce motion artifacts. As was also the case in cats, only the MG muscle was tested since MG and LG have similar architecture and fiber type composition in humans (Johnson et al., 1973; Ward et al., 2009). The cables were connected to a belt-worn wireless transmitter. To record kinematics, light reflective markers were placed on anatomical landmarks (calcaneus, 2nd metatarsal head, lateral malleolus, lateral aspect of shank, lateral epicondyle, lateral aspect of thigh, greater trochanter, anterior/posterior superior iliac spine, acromion process, and head) of both lower limbs and upper body using double-sided adhesive tape.

#### Data collection

Marker positions were recorded using a 3D, 6-camera motion capture system (Vicon Motion Systems Ltd, Oxford, UK) at a sampling rate of 120 Hz. EMG signals from two of the three heads of triceps surae (MG and SO) were band-pass filtered (10–500 Hz) by the pre-amplifiers, transmitted by the belt worn transmitter to a wireless receiver (MyoSystem 1400A, Noraxon Inc., Scottsdale, AZ, USA), amplified by 500×, sampled at 1080 Hz and stored on a computer via Vicon data station for further analysis. The EMG recording system had a constant time delay of 100 ms. Ground reaction forces were collected from one force plate (Bertec Corporation, Columbus, OH, USA) via the Vicon data station at a sampling rate of 1080 Hz.

#### Experimental tasks

***Maximal voluntary contraction***. Participants first performed maximum voluntary contractions (MVC) by ankle extensors using an isokinetic dynamometer (Kin-Com, Isokinetic International, East Ridge, TN, USA) in order to determine the maximum EMG activity of MG and SO. Participants were seated in the Kin-Com chair and the ankle joint axis in the frontal plane was placed in line with the dynamometer arm's axis of rotation. The participant's trunk, thigh, shank, and foot were secured using straps such that flexion angles at the ankle, knee and hip joints were 90°, 0°, and 90° respectively. The participant was instructed to exert maximum ankle plantar flexion against resistance provided by the dynamometer using only the foot in order to target MG and SO muscles. Only EMG activity was recorded during this task. The MVC task was performed under isometric conditions, in which leg joint angles remained unchanged. The subject performed 3 MVC tests with sufficient rest between each contraction. Participants also performed 5 maximum height, countermovement vertical jumps while EMG activity of SO and MG was recorded; subjects were free to swing their arms.

**Figure 3:**
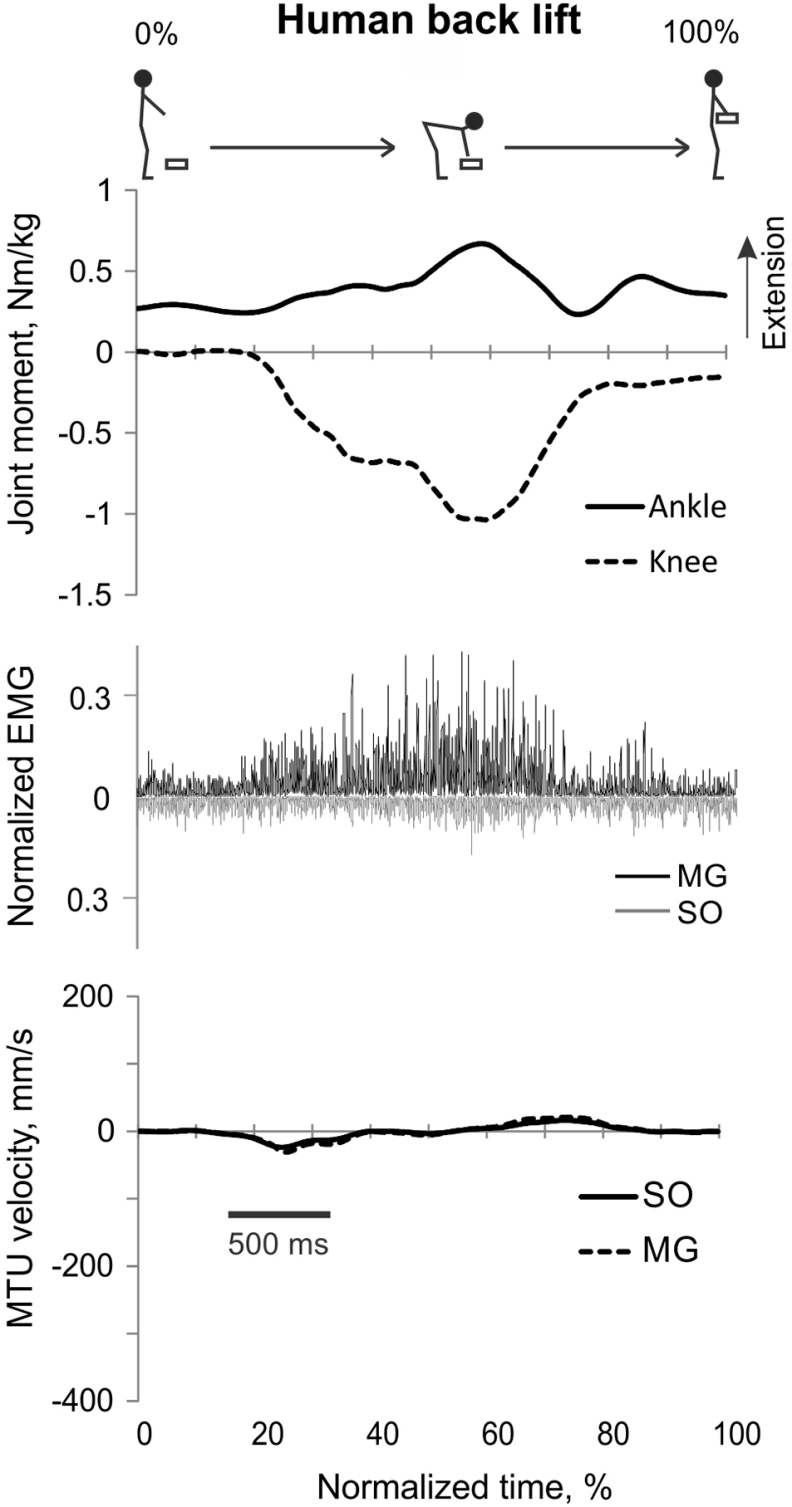
**A representative trial of human back load lifting**. From **top** to **bottom**: Stick figures illustrating the task; normalized resultant muscle moments at the ankle and knee joints (positive values correspond to extension); normalized raw, full-wave rectified EMG activity of MG (positive values) and SO (negative values); and MTU velocity of GA and SO (positive values correspond to lengthening).

**Figure 4:**
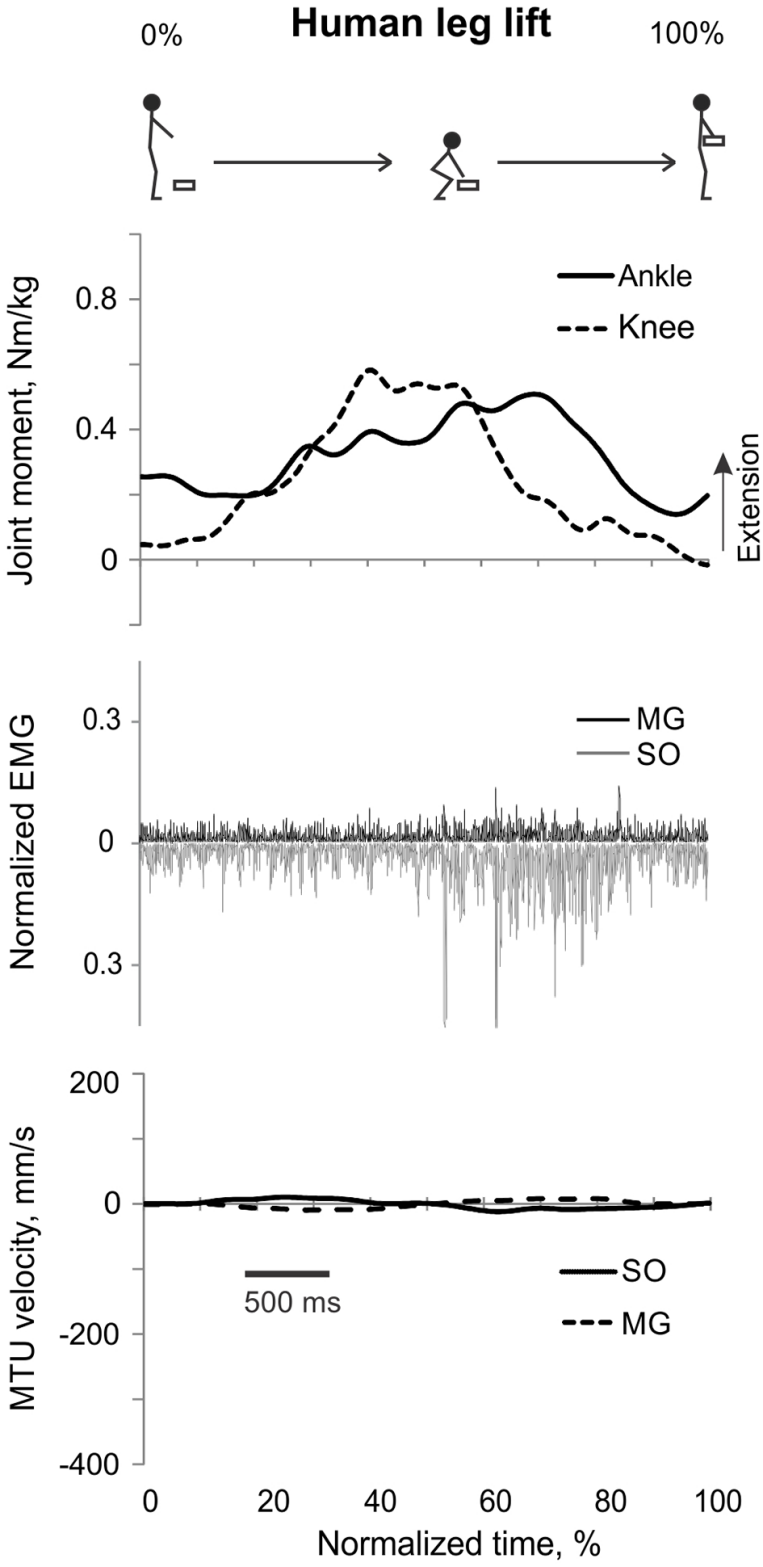
**A representative trial of human leg load lifting**. From **top** to **bottom**: Stick figures illustrating the task; normalized resultant muscle moments at the ankle and knee joints (positive values correspond to extension); normalized raw, full-wave rectified EMG activity of MG (positive values) and SO (negative values); and MTU velocity of MG and SO (positive values correspond to lengthening).

***Load lifting***. Participants completed two types of load lifting, slow tasks (Table 1)—back (straight leg) and leg load lifting—with two loads (5 and 15% of body weight). For the back lift conditions, participants were instructed to reach a plastic box (40 × 32 × 15 cm^3^) filled with sand on the floor in front of the subject from an upright body position and lift the box by extending the back while minimally flexing/extending the knees (Figure 3, stick figures). This task requires production of ankle extension and knee flexion joint moments known to cause high activity of GA (Prilutsky et al., 1998); see also Figure 3. For the leg lifting conditions, participants were instructed to reach the same load from an upright body position by flexing the ankle and knee joints, and then lift the load by extending the leg joints. In contrast to back lifting, this task requires extension joint moments at the ankle and knee (De Looze et al., 1993); see also Figure 4.

In both tasks the load was placed directly in front of the participant at a distance of 20% body height away from the toes and 10% of body height above the ground. One trial consisted of reaching the load on the floor and lifting it up until reaching an upright standing position. Participants performed at least 5 trials for each condition. Before any data were collected, participants were instructed to lift the load at a comfortable, self-selected speed and asked to practice the task for about 1 min before each condition. Participants rested between conditions to minimize fatigue. The order of lifting conditions was randomized.

**Figure 5:**
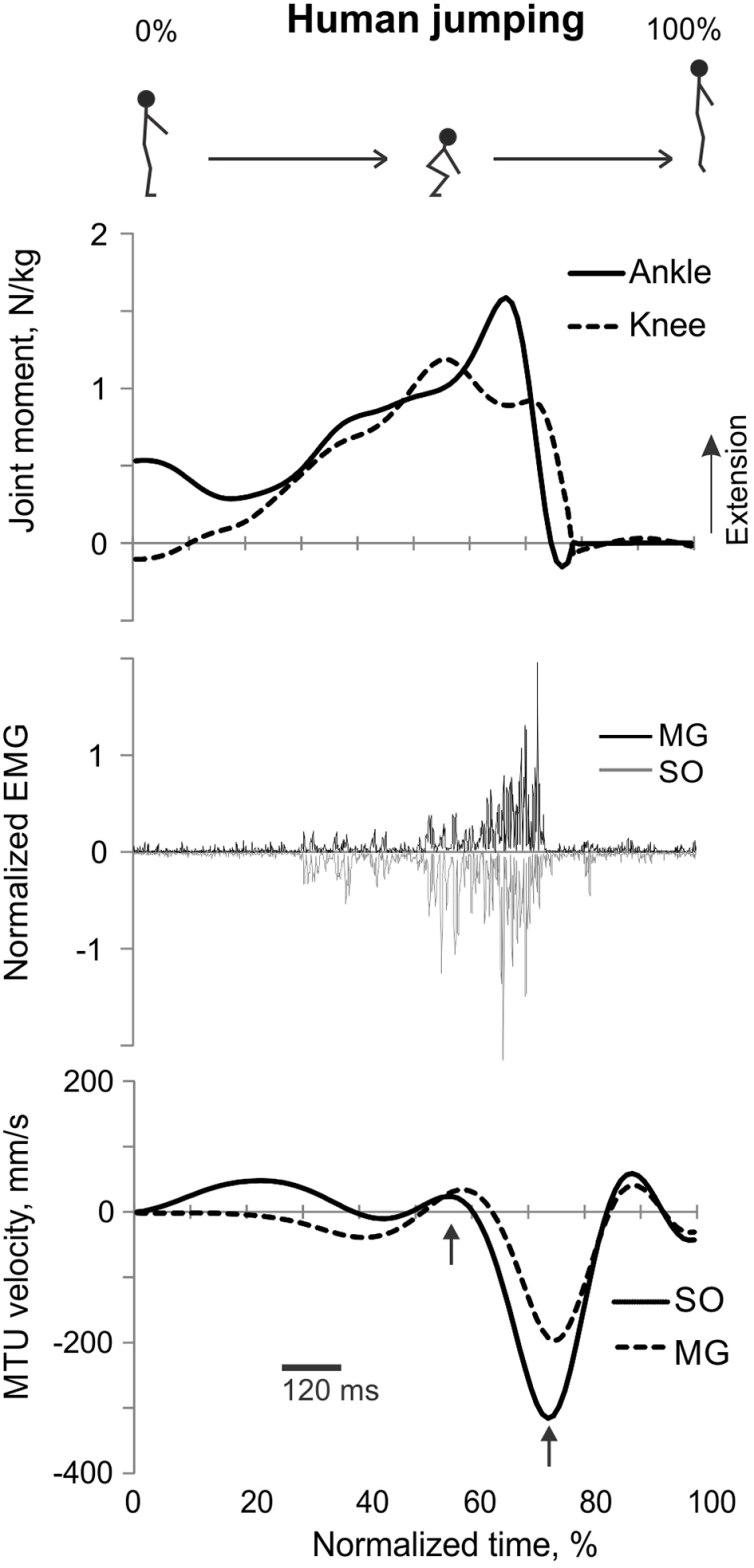
**A representative trial of human jumping**. From **top** to **bottom**: Stick figures illustrating the task; normalized resultant muscle moments at the ankle and knee joints (positive values correspond to extension); normalized raw, full-wave rectified EMG activity of MG (positive values) and SO (negative values); and MTU velocity of MG and SO (positive values correspond to lengthening). Arrows in the bottom panel indicate the time period, for which the mean ankle and knee moments and SO and MG EMG activity were computed.

***Counter-movement vertical jumping***. Participants performed at least 5 trials of a fast task, i.e., a maximum-height counter-movement vertical jump with swinging the arms (Figure 5, stick figures; Table 1). Participants were instructed to jump as high as they could while starting and landing with only the right foot on the force plate. They were given as much rest as needed between jumps.

#### Data analysis and statistics

Recorded leg kinematics and ground reaction forces were used to compute the resultant muscle moments at the right ankle and knee joints using a standard inverse dynamics analysis (e.g., Prilutsky et al., 1998) and a custom computer program written in Matlab (MathWorks, Natick, MA, USA). The kinematic and force data were low-pass filtered at 10 Hz (fourth order, zero-lag Butterworth filter). Body segment inertia parameters were computed for each subject using the regression equations and subject's height and mass (Zatsiorsky, 2002). Smoothed marker coordinates were used to compute joint angles, from which MTU length changes of SO and MG muscles were calculated using the regression equations (Prilutsky and Gregor, 1997). MTU muscle velocities were computed using the method of finite differences.

The load lifting trial time was identified based on the vertical velocity of the head marker. The start of the trial was defined as 0.5 s before reaching the peak of head marker downward vertical velocity; the end of the trial was defined as 0.5 s after reaching the peak of head marker upward vertical velocity. All time dependent mechanical and EMG variables were time normalized to the duration of each lifting trial. The jumping trial onset was defined as 0.25 s before initiation of a downward movement of the shoulder marker; the jumping offset corresponded to 0.25 s after the shoulder marker reached its vertical position at jump onset.

Band-pass filtered EMG signals were full-wave rectified and low-pass filtered (cut-off frequency 10 Hz) to obtain a linear EMG envelope, which was used to compute the mean EMG burst activity of SO and MG during each load lifting and jumping trial defined as described above (see also Figures 3–5). Inspection of the low-pass EMG signals recorded during MVC contractions and vertical jumping revealed that EMG activity for ankle extensor muscles was always greater during jumping than during the MVC task. Therefore, the mean of EMG linear envelope peaks across 5 maximum jumps was used to normalize the EMG activity magnitude of each muscle during all human motor tasks. The ratio (mean SO EMG / mean GA EMG) was computed using normalized EMG values of each load lifting and jumping trial.

EMG low-pass filtered activity, joint moments and MTU velocities were averaged for each percent of the load lifting and jumping time across 5 trials of each condition and participant and then across participants since all subjects showed similar trends. In addition, the mean ankle and knee joint moments, mean SO and MG EMG and the SO EMG/MG EMG ratio were computed for the time periods of each load lifting corresponding to the ankle extension-knee flexion joint moment combination (during back lifting, Figure 3, Table 1) and to the ankle extension-knee extension joint moment combination (during leg lifting, Figure 4, Table 1). The same analysis was performed for jumping, however the period for determining the mean moments and EMG was the time between the two peaks of SO MTU velocity corresponding to approximately 55 and 75% of the jump trial when highest SO and MG activity and joint moments occurred (Figure 5, arrows).

A linear mixed model analysis (IBM SPSS Statistics v20 software, Chicago, IL, USA) was used to test the hypothesis that the SO EMG/MG EMG ratio would be lower during a slow task of back load lifting (at the ankle extension-knee flexion joint moment combination) than during a slow task of leg load lifting or a fast task of vertical jumping (at the ankle extension-knee extension joint moment combination). One-sample *t*-tests were performed to determine whether the SO EMG/MG EMG ratio for each task was significantly different from 1. The linear mixed model analysis was also used to examine effects of joint moment combination and movement speed in all studied tasks on the mean SO and MG EMG. Significance for all tests was set at an alpha level of 0.05.

## Results

### Cat experiments

#### Paw shake response

**Figure 6:**
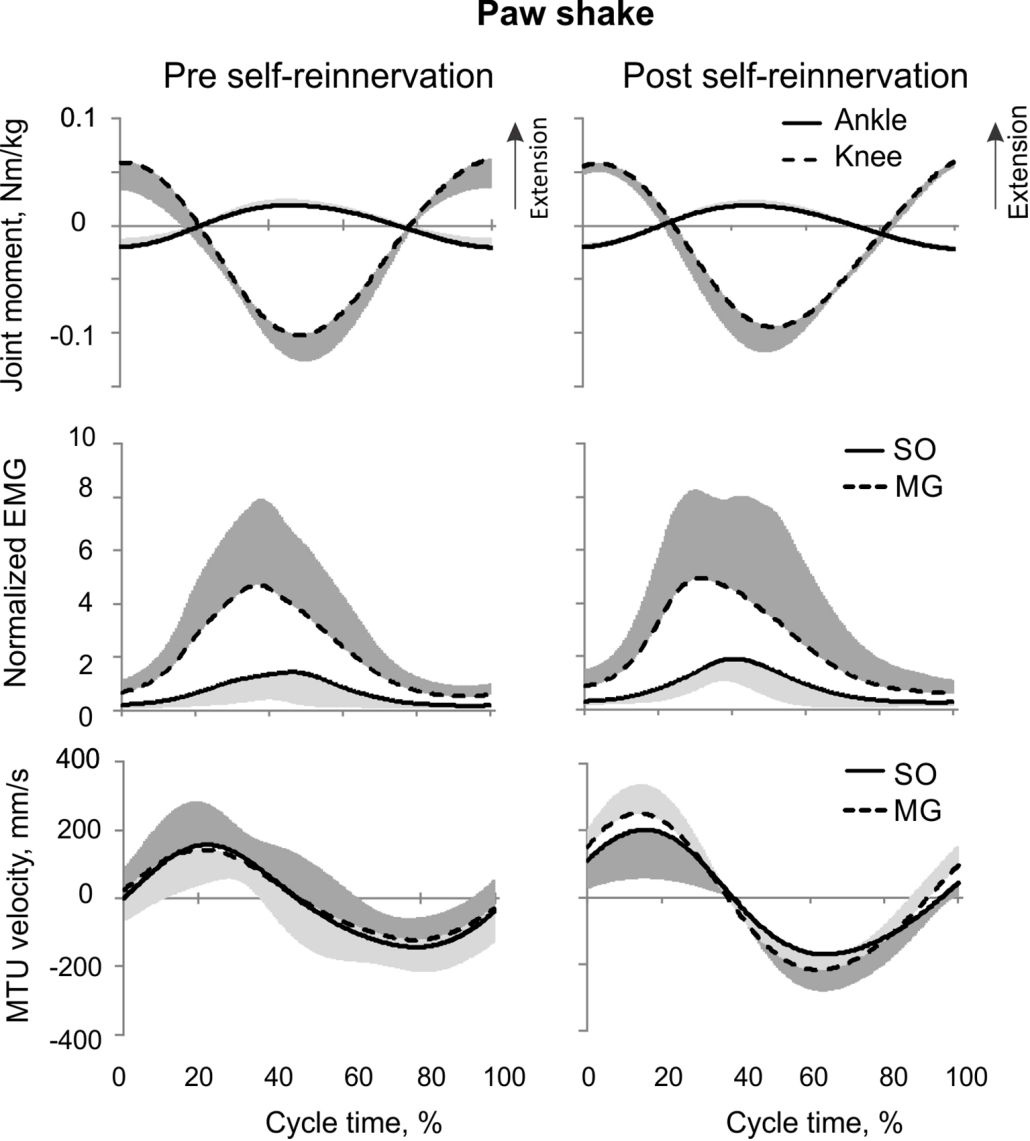
**Mean (±*SD*) of normalized muscle moments at the ankle and knee joints (top), low-pass filtered normalized EMG of MG and SO (middle), and MTU velocity of MG and SO (bottom) during the paw shake cycle**. The mean and *SD* values for pre- and post-reinnervation conditions (left and right panels, respectively) were computed across all studied paw shake cycles (at least 5 per cat) and cats.

**Figure 7:**
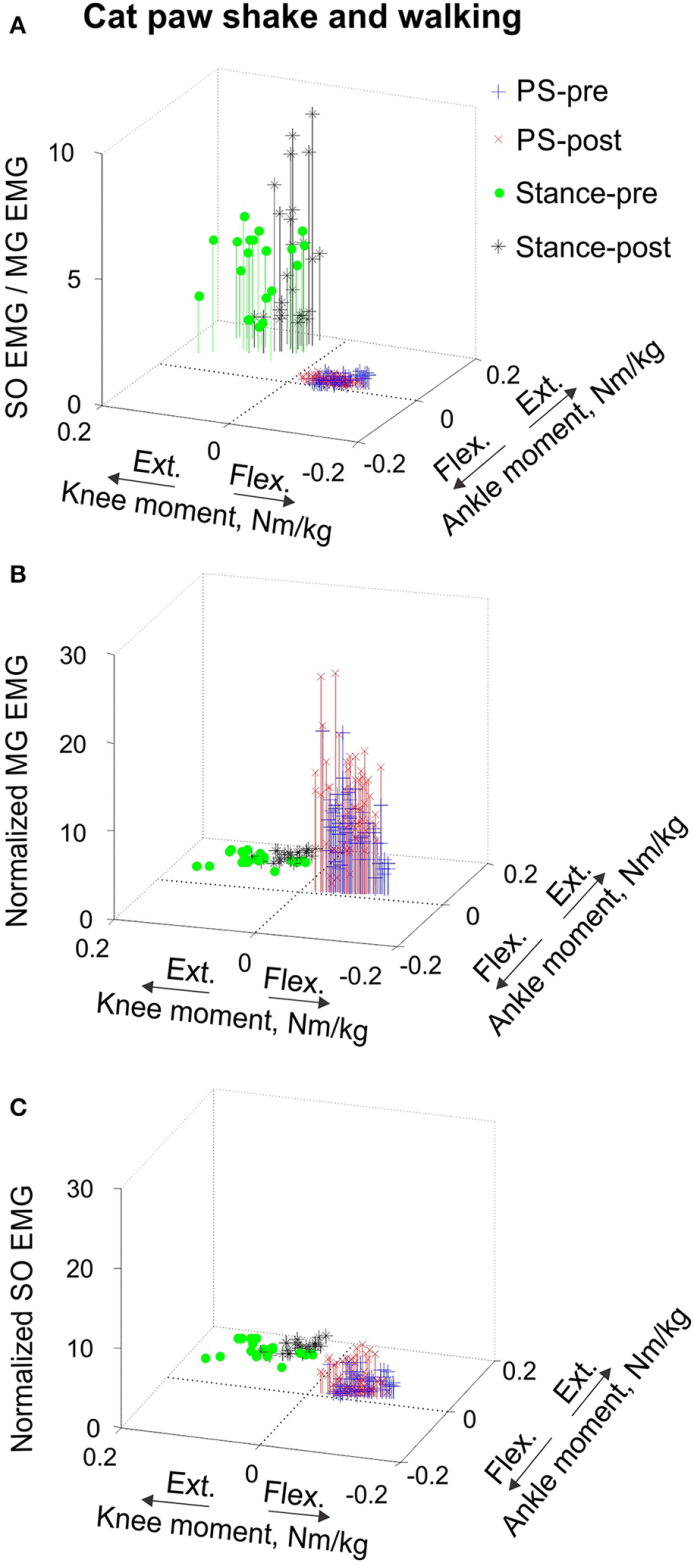
**Mean SO EMG/MG EMG ratio (A), mean normalized MG EMG activity (B) and mean normalized SO EMG activity (C) as functions of the mean normalized ankle and knee resultant muscle moments during paw shake responses and stance of walking in the cat**. Each symbol corresponds to the mean normalized EMG activity plotted vs. the respective mean normalized ankle and knee moment in a given movement cycle. During paw shake responses, SO and MG EMG activity bursts occurred at the ankle extension-knee flexion joint moment combination (purple and red crosses designate paw shake cycles recorded pre and post SO-GA self-reinnervation; *n* = 62 and *n* = 62, respectively). During the stance phase of walking, SO and MG EMG bursts occurred at the ankle extension-knee extension joint moment combination (green circles and black stars correspond to stance phases recorded pre and post SO-GA self-reinnervation; *n* = 20 and n = 25, respectively).

Typical paw shake episodes in intact cats consisted of 4–7 cycles of fast hindlimb oscillations with frequencies between 8 and 12 Hz (Figure 1). During steady state paw oscillations, which occurred typically in the middle of paw shake episodes, the ankle extension and knee flexion joint moments, MG and SO EMG bursts and MTU positive velocity (lengthening) occurred at approximately the same time period in the cycle (Figures 1, 6). In the other half of the paw shake cycle, the ankle flexion and knee extension moments were produced simultaneously; during this period SO and MG muscles had no or low activity and were mostly shortening (Figures 1, 6). MTU lengthening and shortening velocity peaks were reaching values of about ± 150 mm/s on average, which exceeded peak lengthening and shortening MTU velocities during cat level walking up to 3 times, respectively (Figures 1, 6; Cronin et al., 2013) and corresponded to approximately 81 and 47% of the maximum shortening velocities of cat SO and MG (Table 1; Spector et al., 1980). Before reinnervation, SO EMG bursts were very short and their mean magnitude was higher than that during level walking [*F*_(1, 77)_ = 14.08, *p* < 0.001; Figures 1, 2, **12B**]; the mean MG EMG was over 10 times higher during paw shake than during level walking [*F*_(1, 77)_ = 135.38, *p* < 0.001; Figures 1, 2, **12A**). As a result, the SO EMG/MG EMG ratio was much smaller during paw shake response (0.26 ± 0.18), at the ankle extension-knee flexion moment combination, than during stance of level walking, at the ankle extension-knee extension moment combination [3.23 ± 1.34, *F*_(1, 79)_ = 334.75, *p* < 0.001; Figures 7A, **11A,B**]. The ratio was also statistically smaller than 1 (one-sample *t*-test; *n* = 62, *p* < 0.001).

Reinnervation of SO-LG or MG-LG muscle combinations did not cause apparent changes in patterns of joint moments, EMG activity or MTU velocities. Peak EMG bursts of SO and MG after self-reinnervation also occurred in the vicinity of the ankle extension and knee flexion moment peaks, which approximately coincided with muscle lengthening (Figure 6). As in intact cats, the mean SO EMG burst magnitude was low in comparison with the mean MG EMG magnitude. The mean magnitude of MG EMG burst increased after self-reinnervation [*F*_(1, 119)_ = 6.75, *p* = 0.011; **Figure 12A**], although the SO EMG/MG EMG ratio did not change significantly [*F*_(1, 122)_ = 0.51, *p* = 0.476, Figures 7A, **11A,B**).

#### Level walking

EMG activity bursts of SO and MG muscles in intact cats occurred mostly during the stance phase, when the moments at the ankle and knee joints were extension and peaks of MTU lengthening and shortening velocities of SO and MG were much lower than during the swing phase (Figure 2B) or paw shake responses (Figures 1, 6, Table 1). The mean normalized EMG magnitude of SO was 0.77 ± 0.12 and that of MG was 0.30 ± 0.17 during the stance phase of walking (Figures 7B,C, **12A,B**). The SO EMG/MG EMG ratio (3.23 ± 1.34) was several times higher during stance of walking than during paw shake response [*F*_(1, 79)_ = 334.75, *p* < 0.001] and exceeded 1 (one-sample *t*-test; *n* = 20, *p* < 0.001); (Figures 7A, **11A,B**).

Self-reinnervation of SO and GA muscles did not cause changes in the normalized mean activity of SO and MG during stance of walking [*F*_(1, 40)_ = 2.57, *p* = 0.117 and *F*_(1, 39)_ = 0.142, *p* = 0.707, respectively; **Figures 12A,B**]. The SO EMG/MG EMG ratio on average did not change either [*F*_(1, 39)_ = 1.64, *p* = 0.208; **Figure 11B**), although in two cats with MG-LG self-reinnervation the ratio significantly increased [*F*_(1, 28)_ = 192.44, *p* < 0.001; **Figure 11A**]. Overall, self-reinnervation of SO and GA did not change the SO EMG/MG EMG ratio, which was still several times greater during stance of walking than paw shake response (Figures 7A, **11B**).

### Human experiments

#### Back load lifting

**Figure 8:**
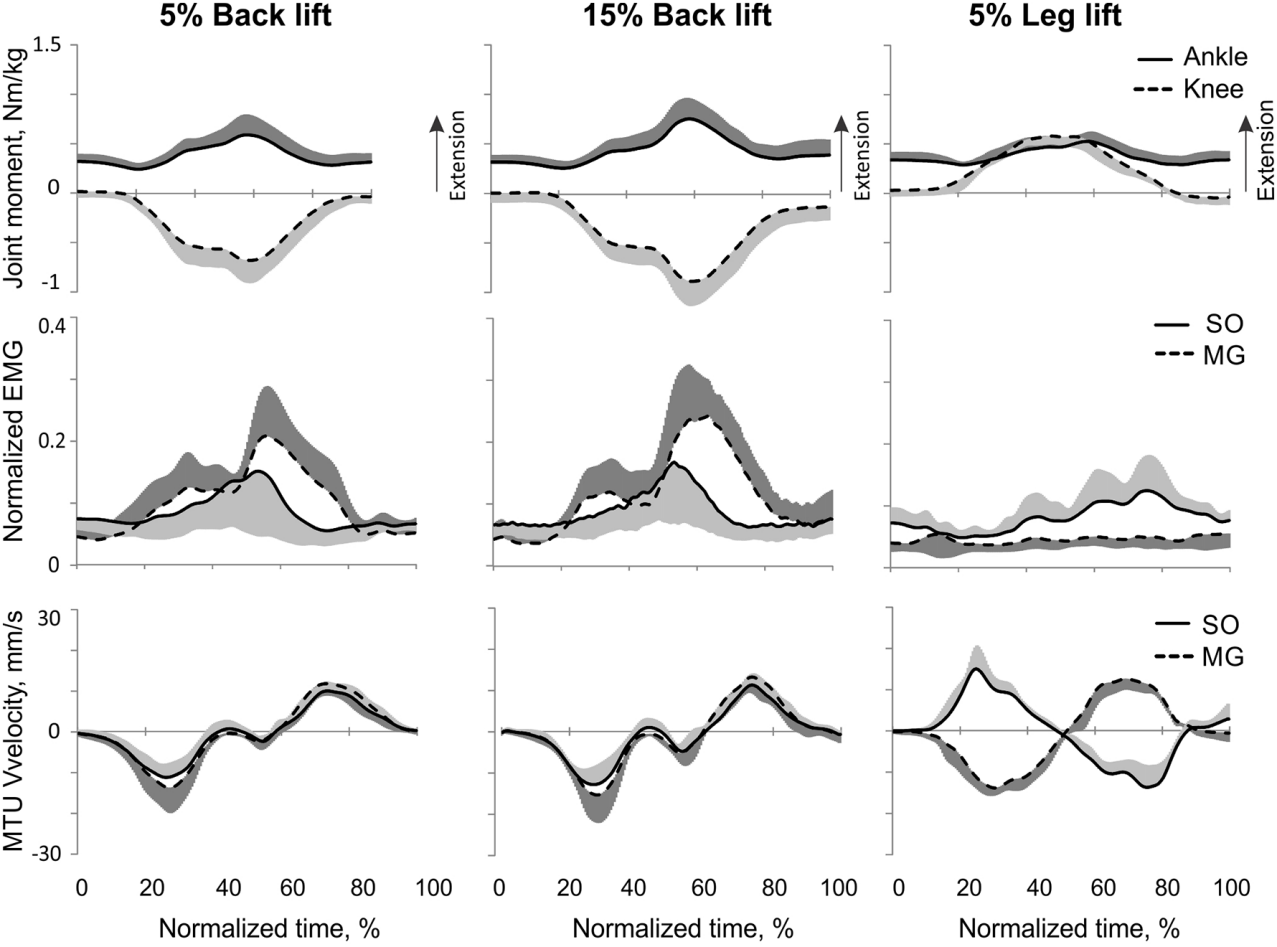
**Mean (±*SD*) normalized ankle and knee joint moments (top traces), low-pass SO and MG EMG activity (middle traces), and MTU SO and MG velocity (bottom traces) during human back load lifting (left column, load 5% body weight; middle column, load 15% of body weight) and leg load lifting (load 5% body weight)**. Positive moments and MTU velocity designate joint moment extension and MTU lengthening, respectively.

During back load lifting, ankle extension and knee flexion moments were produced simultaneously, MG EMG activity was sharply modulated in parallel with the magnitudes of ankle and knee moments while the SO EMG magnitude increased initially and then decreased earlier than MG EMG (Figure 8). Peaks of MTU lengthening and shortening velocities of SO (11.3 ± 2.7 and −12.9 ± 4.4 mm/s, respectively) and MG (13.2 ± 3.9 and −15.3 ± 6.6 mm/s, respectively) were very low compared to the corresponding MTU velocity peaks during human walking (300 and 350 mm/s), respectively [(Cronin et al., 2013); Figures 3, 8] or to the maximum shortening velocities of human SO and MG (Table 1; Prilutsky, 2000b). Patterns of ankle and knee joint moments, SO and MG EMG activity, and MTU velocities were generally similar between 5- and 15%-loads (Figure 8) with slightly greater magnitudes of joint moments and EMG activity reaching statistically significant differences only for the mean MG EMG [*F*_(1, 47)_ = 47.10, *p* = 0.015; **Figure 12C**]. The SO EMG/MG EMG ratios during back load lifting were 0.85 ± 0.40 and 0.92 ± 0.31 for 5 and 15%, respectively, and they were not statistically different [*F*_(1, 47)_ = 0.002, *p* = 0.966, **Figure 11C**]. The SO EMG/MG EMG ratios combined together from the two tasks were statistically less than 1 (one-sample *t*-test; *n* = 52, *p* = 0.021; **Figure 11C**).

#### Leg load lifting

During leg load lifting, with the extension muscle moments at the ankle and knee, SO EMG was modulated roughly in parallel with ankle extension moment while MG EMG was relatively constant and low (Figures 4, 8). MTU velocity changes of SO and MG during leg load lifting were opposite; SO MTU was lengthening during the first half of the movement (squatting) and then shortening during load lifting in the next half (extending legs), whereas MG MTU was shortening first (due to much larger knee flexion than ankle flexion) and then lengthening (Figures 4, 8). The mean normalized EMG activity of SO (0.080 ± 0.017) and MG (0.047 ± 0.014) was lower [*F*_(1, 48)_ = 9.60, *p* = 0.003 and *F*_(1, 48)_ = 258.23, *p* < 0.001, respectively] compared to the back lift values (**Figures 12C,D**). The SO EMG/MG EMG ratio (1.88 ± 0.64) was almost 2 times higher during leg lift than back lift [*F*_(1, 48)_ = 163.41, *p* < 0.001, **Figure 11C**] and it was significantly greater than 1 (one-sample *t*-test, *n* = 29, *p* < 0.001).

#### Counter-movement vertical jumping

**Figure 9:**
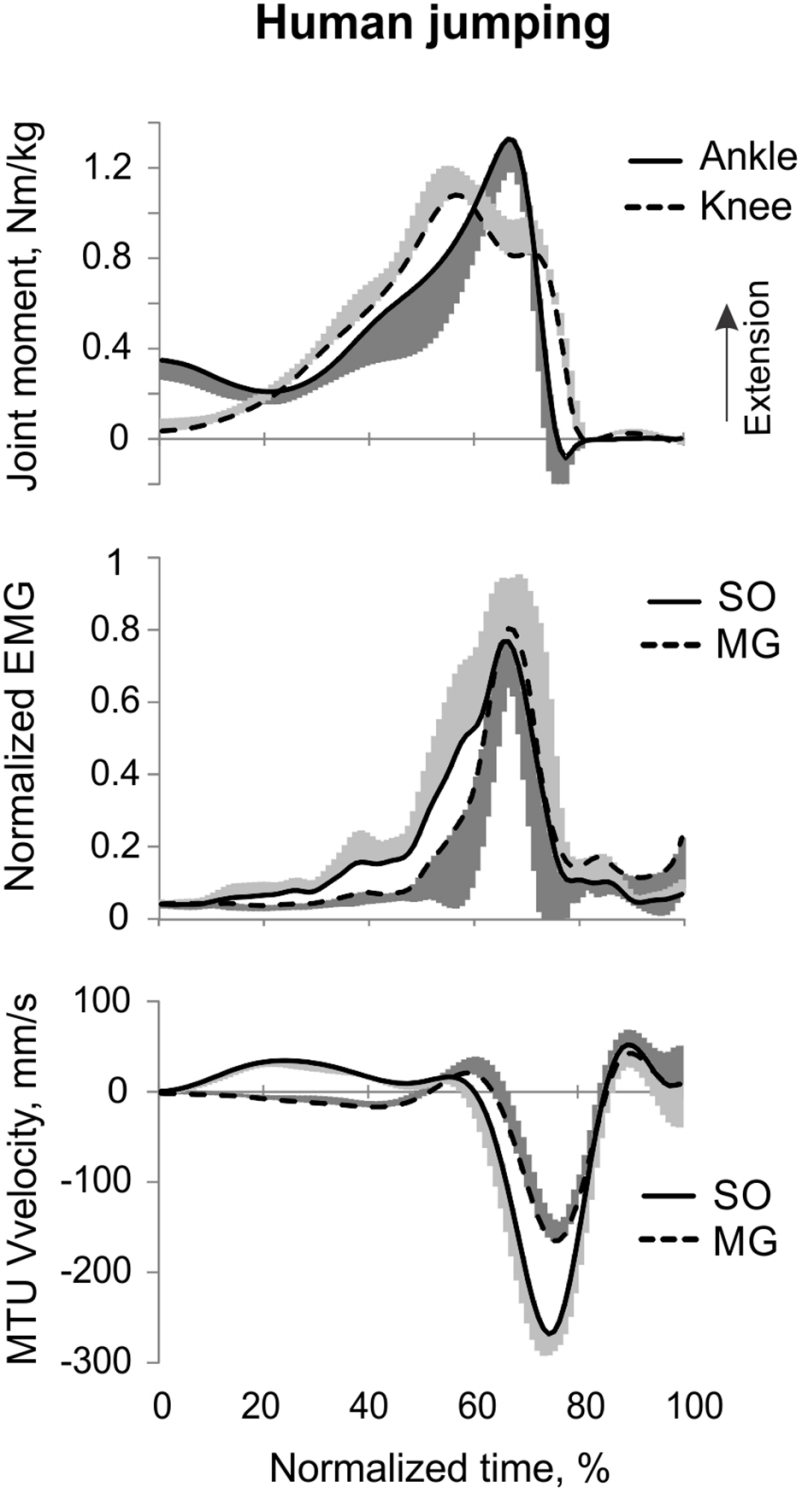
**Mean (±*SD*) normalized ankle and knee joint moments (top traces), low-pass SO and MG EMG activity (middle traces), and MTU SO and MG velocity (bottom traces) during human jumping across all subjects (*n* = 5)**. Positive moments and MTU velocity designate joint moment extension and MTU lengthening, respectively.

**Figure 10:**
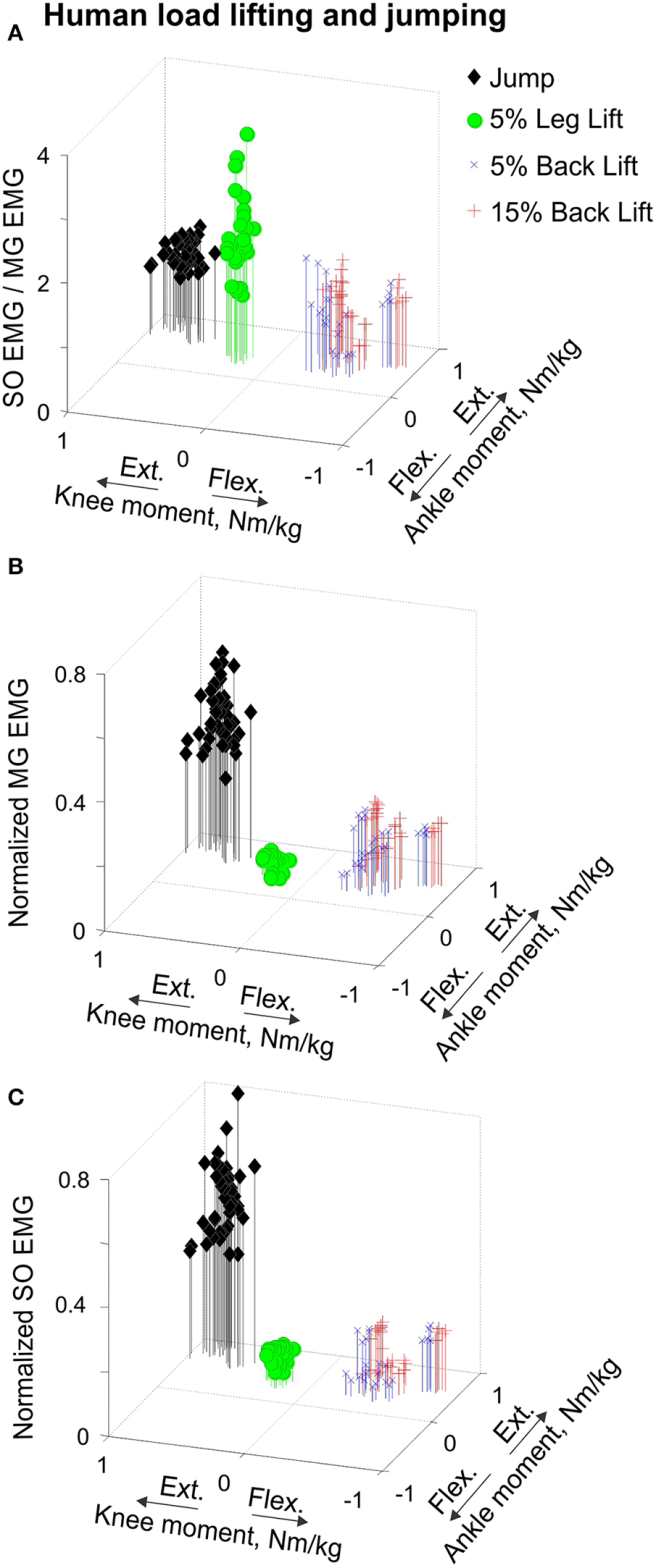
**Mean SO EMG/MG EMG ratio (A), normalized mean MG EMG activity (B) and normalized mean SO EMG activity (C) as functions of the mean ankle and knee resultant muscle moments during human load lifting and jumping**. Each symbol corresponds to the mean EMG activity plotted vs. the respective mean ankle and knee moment obtained for a single load lifting or jumping trial. During back load lifting, SO and MG EMG activity occurred at the ankle extension-knee flexion joint moment combination (blue and red symbols designating lifting 5 and 15% body weight loads, *n* = 25 and *n* = 28, respectively). During leg load lifting and jumping, SO and MG EMG activity occurred at the ankle extension-knee extension joint moment combination (green circles, *n* = 29 and black diamonds, *n* = 40, respectively).

**Figure 11:**
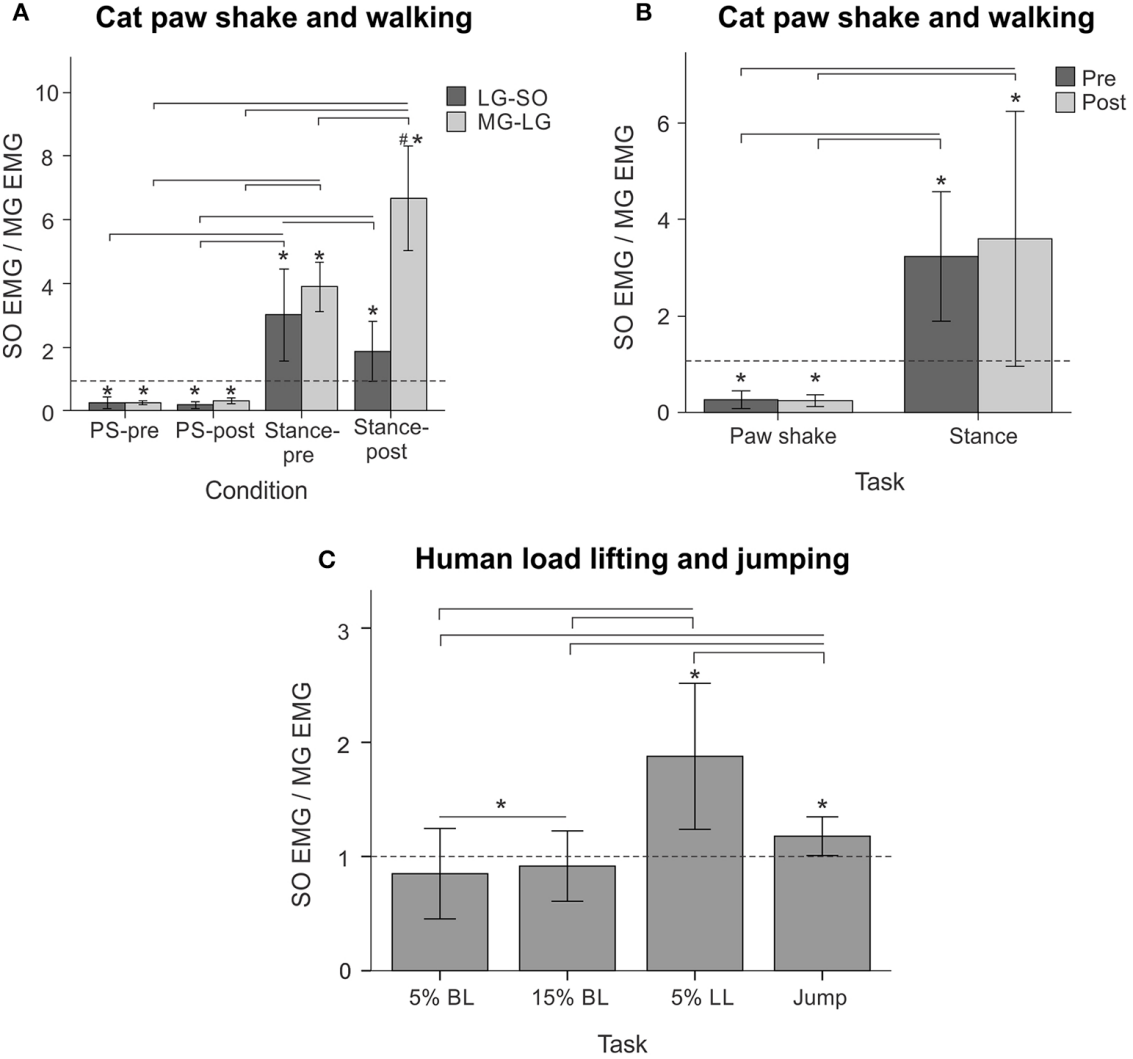
**SO EMG/MG EMG ratio (mean ± *SD*) during different motor behaviors performed by cats and humans**. Horizontal dashed lines indicate the ratio value of 1. **(A–B)** SO EMG/MG EMG ratio during cat paw shake responses and stance of walking pre and post self-reinnervation of SO-LG (in 4 cats) and MG-LG (in 2 cats) muscles. Horizontal bars with brackets indicate statistical significance (*p* < 0.05) between experimental conditions (motor tasks), symbol # indicates a significant difference between pre and post self-reinnervation, asterisks indicate statistically significant difference of the ratio from 1. **(C)** SO EMG/MG EMG ratio during human back load lifting (5 and 15% body weight), leg load lifting (5% body weight) and vertical jumping. Horizontal bars with brackets indicate statistical significance (*p* < 0.05) between experimental conditions (motor tasks), asterisks indicate statistically significant difference of the ratio from 1.

**Figure 12:**
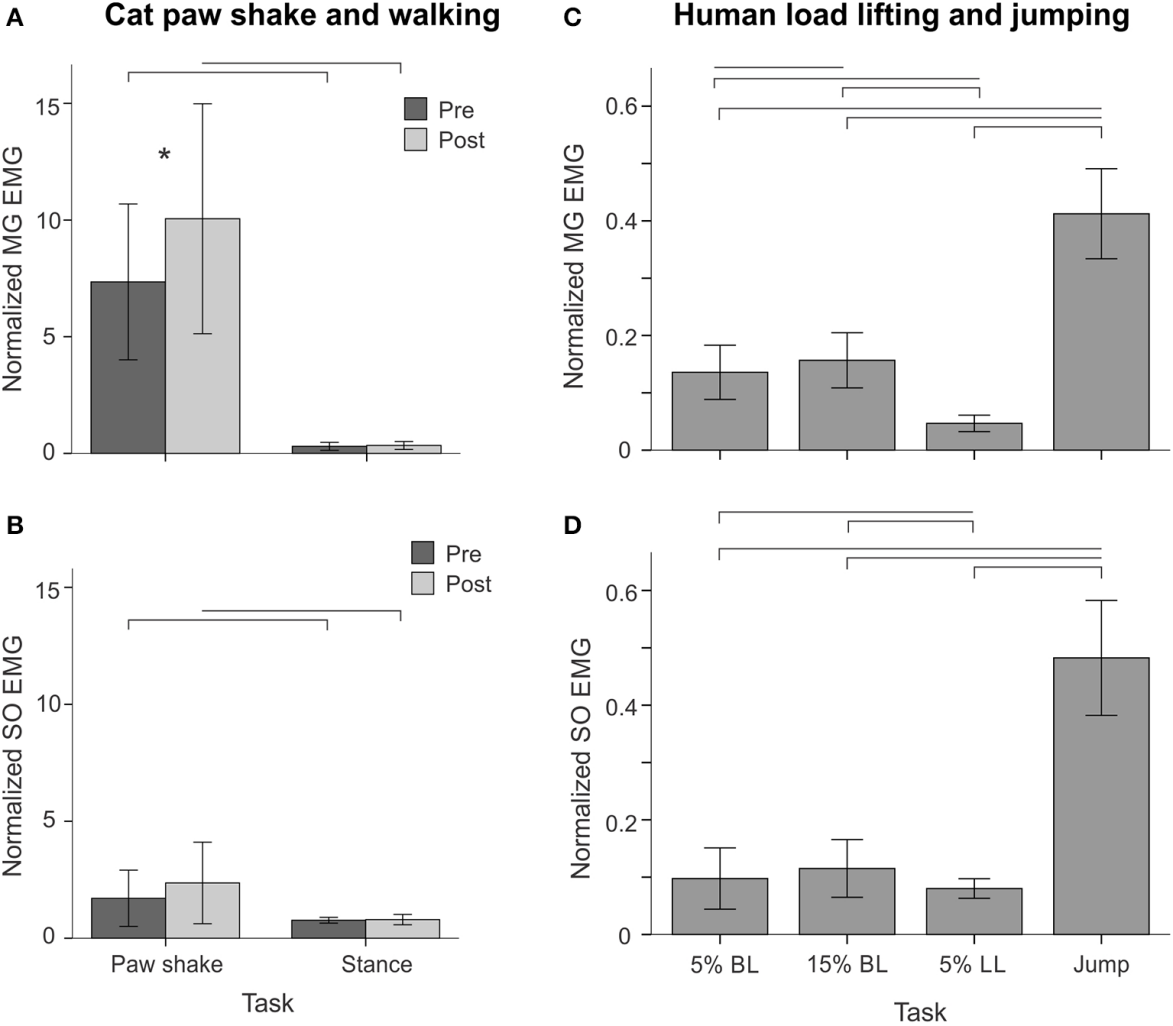
**Normalized MG and SO EMG activity (mean ± *SD*) during different motor behaviors performed by cats and humans**. **(A,B)** MG and SO EMG during paw shake responses and stance of walking pre and post self-reinnervation of SO-LG muscles in 4 cats and MG-LG muscles in 2 cats. Horizontal bars show statistical significance (*p* < 0.05) between experimental conditions (motor tasks), asterisk indicates a significant difference between pre and post self-reinnervation. **(C, D)** Normalized MG and SO EMG during human back load lifting (5 and 15% body weight), leg load lifting (5% body weight) and vertical jumping. Horizontal bars indicate statistically significant difference (*p* < 0.05) between experimental conditions (motor tasks).

The resultant joint moments at the ankle and knee were both extension during the counter-movement phase (from ~0 to 60% of the jump period) and push-off phase (from ~60 to 80%) of jumping until the lift-off at ~80% of the jump period (Figures 5, 9). During the counter-movement phase, SO MTU velocity was positive indicating muscle lengthening, while MG MTU was shortening with a low negative velocity due to a greater flexion at the knee than at the ankle. Both muscles showed high peaks of shortening velocities during the push-off phase (155 and 24% of the estimated maximum shortening velocities of SO and MG (Figure 9, Table 1; Prilutsky, 2000b) with a lower velocity peak in MG due to opposite MTU length changes caused by ankle and knee extensions. EMG activity of SO and MG started to rise in the middle of the counter-movement and reached very high values in both muscles during the push-off phase (Figures 5, 9). The normalized mean EMG activity of SO (0.482 ± 0.100) and MG (0.412 ± 0.079) during the push-off phase was significantly greater than during the back and leg load lifting tasks [*F*_(3, 121)_ = 298.2, *p* < 0.001 and *F*_(3, 121)_ = 290.7, *p* < 0.001, respectively; Figures 10B,C, **12C,D**]. The SO EMG/MG EMG ratio during jumping (1.18 ± 0.17) was significantly greater than for the back load lifting tasks [*F*_(3, 121)_ = 39.5; *p* = 0.008]. In contrast, the ratio was significantly lower in jumping compared to leg load lifting [*F*_(3, 121)_ = 39.5, *p* < 0.001; Figures 10A, 11C). The SO EMG/MG EMG ratio for jumping was significantly greater than 1 (one-sample *t*-test, *n* = 43, *p* < 0.001).

## Discussion

### Comparison with previous studies

Results obtained in this study on mechanics and muscle activity of paw shake responses and walking in intact cats, as well as load lifting and vertical jumping in humans are in good agreement with the literature. Several reports described a relative inhibition of SO EMG or force with respect to MG EMG or force during paw shake responses (Smith et al., 1980, 1985; Abraham and Loeb, 1985; Fowler et al., 1988; Hodson-Tole et al., 2012). During steady-state paw shakes, triceps surae EMG activity occurred during MTU stretch, as in our study (Figures 1, 6), in phase with the extremely high activity of muscle velocity-sensitive group Ia afferents (Prochazka et al., 1977, 1989). Although, previous kinetic analysis of paw shake responses focused primarily on the interactive motion-dependent moments at the joints (Hoy et al., 1985; Hoy and Zernicke, 1986; Smith and Zernicke, 1987), selected illustrations of the resultant muscle moments at the ankle and knee in those studies did indicate the ankle extension-knee flexion muscle moment combination during one half of the paw shake cycle (see Figure 7, muscle moments in Hoy and Zernicke, 1986).

SO and MG EMG activity, resultant muscle moments at the ankle and knee, and MTU length changes during cat level walking have been well documented in the literature (e.g., Goslow et al., 1973; Fowler et al., 1993; Gregor et al., 2006; Prilutsky et al., 2011) and are in good agreement with our results. Reinnervated ankle extensors in the cat have been reported to recover their EMG activity patterns and magnitude 12–13 weeks after nerve cut and surgical repair (O'Donovan et al., 1985; Gregor et al., 2003; Pantall et al., 2012) as also shown in our study (Figures 6, 12A,B).

Patterns and peak values of the resultant muscle moments at the ankle and knee during back and leg load lifting and the corresponding MG EMG activity patterns obtained in this study Figures 3, 4, 8) are in good agreement with other studies (De Looze et al., 1993; Prilutsky et al., 1998). For example, the ankle extension-knee flexion and ankle extension-knee extension moment combinations occurred during back and leg load lifting, respectively, with much reduced MG EMG during leg load lifting compared with back load lifting. We have not found studies in which SO EMG was measured and compared with MG EMG during load lifting.

Ankle and knee resultant muscle moments, MTU velocities and EMG activity patterns of SO and MG obtained in our study during vertical jumping (Figures 5, 9) are generally similar to those reported in other studies. For example, the ankle and knee moments are extension and reach their peak values during the push-off phase of jumping; the SO MTU peak shortening velocity is higher than that of MG MTU; and SO and MG are highly active during vertical jumping (e.g., Bobbert et al., 1986; Bobbert and Van Ingen Schenau, 1988; Kurokawa et al., 2001).

### Study hypotheses

This study tested two major explanations for the relative inhibition of the slow-twitch head of triceps surae SO with respect to a fast-twitch head MG. The explanation that this inhibition requires high movement velocities and mediated by the stretch velocity-sensitive spindle Ia afferents was tested by comparing the SO EMG/MG EMG ratio between fast tasks of cat paw shaking and human vertical jumping and slow tasks of cat walking and human load lifting (Table 1). In addition, the SO EMG/MG EMG ratio was determined during paw shaking and stance of walking in intact cats and cats without stretch reflex in SO and GA removed by self-reinnervation of these muscles. The second explanation that the relative SO inhibition depends on the ankle-knee combination of joint moments and does not require high muscle velocities was tested by comparing the SO EMG/MG EMG ratio between tasks with the ankle extension-knee flexion combination of joint moments (fast cat paw shaking and slow human back load lifting) and with the ankle extension-knee extension moment combination (fast human vertical jumping and slow human leg load lifting and cat walking, Table 1).

The obtained results do not support the hypothesis that SO inhibition requires high movement velocities and is mediated by muscle Ia velocity-sensitive afferents. Removal of stretch velocity-dependent afferent feedback from SO-LG in 4 cats and MG-LG in 2 cats by their self-reinnervation (Cope and Clark, 1993; Cope et al., 1994; Alvarez et al., 2011; Bullinger et al., 2011) did not affect the SO EMG/MG EMG ratio (Figure 11B) or EMG activity of these muscles in relation to the resultant muscle moments at the ankle and knee (Figure 6). Although intact synergists of self-reinnervated SO and MG, such as plantaris, can still supply SO and MG motoneurons with velocity-dependent afferent input (Eccles et al., 1957; Nichols, 1994), this input is expected to be dramatically diminished by self-reinnervation of SO and GA. In addition, we observed the relative SO inhibition (the SO EMG/MG EMG ratio was below 1 in nearly isometric task of back load lifting (Figures 8, 11C), in which velocity-dependent afferent input is expected to be very low. Furthermore, we found no SO inhibition with respect to MG (the SO EMG/MG EMG ratio above 1, Figure 11C) and a very high mean SO activity (Figure 12D) during a fast task of human vertical jumping. Thus, the first hypothesis cannot explain the relative inhibition of SO during paw shake responses in cats with self-reinnervated SO and GA and during slow back load lifting tasks in humans, neither it can explain the high SO EMG and lack of the relative inhibition of SO during fast vertical jumping.

The second hypothesis that relative SO inhibition depends on the ankle-knee moment combination and does not require high movement velocities received strong support in this study. The SO EMG/MG EMG ratio was lower than 1 at the ankle extension-knee flexion moment combination (fast cat paw shaking and slow human back load lifting), whereas the ratio was greater than 1 (i.e., no SO inhibition) at the ankle extension-knee extension moment combination (fast human jumping, slow cat walking, and slow human leg load lifting; Figure 11), irrespective of MTU velocities (Figures 6, 8, 9; Table 1).

Our rejection of hypothesis 1 and support for hypothesis 2 are based on comparisons of SO and MG EMG activities and functions across two different species, cats and humans. Although morphology, architecture and size of SO and MG muscles may differ substantially from one species to another, e.g., dogs do not have SO (Spoor and Badoux, 1989) and SO in horses is very small to contribute significantly to ankle extension (Meyers and Hermanson, 2006), the difference in properties of SO and MG muscles between cats and humans seems sufficiently small and that justifies our comparisons. Specifically, muscle volumes of SO and MG, determined as the product of the mean muscle fiber length and physiological cross-sectional area, constitute 10 and 21% of total volume of all ankle extensors, respectively, in cats (Sacks and Roy, 1982) and 45 and 21% in humans (Ward et al., 2009). The SO is a slow-twitch muscle in cats and humans—the percentage of slow-twitch muscle fibers is 100% in cats (Ariano et al., 1973) and 88% in humans (Johnson et al., 1973), whereas the MG is a predominantly fast-twitch muscle in cats (25% of slow-twitch fibers) and a mixed muscle in humans, 51%.

### Potential mechanisms and functional significance of differential activity of SO and MG

Differential activity of individual heads of triceps surae has been first observed during paw shake responses in the cat (Smith et al., 1980). It is characterized by an increased EMG activity of GA and a relatively low SO EMG. The relative inhibition of SO activity during slow human movements is shown for the first time in the present study. Note that in the current study, the mean SO EMG during fast cat paw shake responses and slow human back load lifting was higher than during cat stance of walking (Figure 12B) and human leg load lifting (Figure 12D), respectively, although the SO EMG/MG EMG ratio was much lower during paw shake responses (Figures 11A,B) and back load lifting (Figure 11C). The differences in movement speed cannot consistently explain this behavior, as discussed above. Although it may seem counterintuitive, activating slow-twitch muscles like SO during high-speed movements may be mechanically advantageous for achieving high movement velocities (Holt et al., 2014).

The fact that (1) task demands for paw shake responses and back load lifting require the simultaneous production of ankle extension and knee flexion moments and that (2) the mean MG EMG activity in these tasks is much higher than during cat stance of walking and human leg lifting (the latter two tasks require ankle extension and knee extension moments) (Figures 12A,C) is consistent with the idea that increasing the relative contribution of the two-joint MG to production of ankle extension and knee flexion moments simultaneously is mechanically advantageous. The task specific increase in MG activity could involve excitatory inputs from central spinal circuitry comprising both the extensor and flexor half centers of a central pattern generator (Perret and Cabelguen, 1980; Prilutsky, 2000a; Shevtsova et al., 2014) and possibly proprioceptive feedback other than velocity-dependent one (Klishko et al., 2011). The simultaneous excitatory inputs to the MG motoneuron pool from extensor and flexor spinal centers, explaining higher MG activity, is also consistent with the relatively lower activity of SO, whose motoneurons presumably receive central excitatory input only from the extensor center.

During vertical jumps, both SO and MG demonstrated the highest mean EMG magnitude among all human tasks (Figures 12C,D) and the ratio SO EMG/MG EMG exceeding 1 (Figure 11C). These results likewise can be explained by a strong excitatory input to motoneuronal pools of both muscles from an extensor center, whereas the relatively lower MG activity with respect to SO could be caused by inhibitory influences on MG motoneurons. A spinal pathway that could provide such inhibition is the force-dependent inhibitory pathway from the quadriceps, knee extensor muscles that are highly active during jumping (Bobbert and Van Ingen Schenau, 1988), to MG (Wilmink and Nichols, 2003). This inhibition along with the length-dependent excitation from the vastii, one-joint knee extensors, to SO (Wilmink and Nichols, 2003) appear to be functionally appropriate during motor tasks with ankle extension-knee extension moment combinations, as in vertical jumping and leg load lifting, because they act to reduce the MG antagonistic action at the knee joint and the MG contribution to the ankle extension moment. Another potential mechanism for MG inhibition could be muscle length related—MG activity has been shown to decrease and SO activity to increase during production of ankle extension moments while the knee joint is flexing causing shortening of MG fascicles (Lauber et al., 2014). The latter mechanism does not seem to influence substantially MG activity in this study as similar maximum knee flexion angles during leg lifting and vertical jumping (82 ± 14 and 89 ± 8°, respectively) produced dramatically different MG and SO EMG activities (Figures 12C,D).

Although we did not find evidence of a decrease in the mean SO activity during paw shake responses or back load lifting compared to walking or leg load lifting (Figures 12B,D, respectively), we cannot exclude inhibitory influences on SO motoneurons from central or peripheral sources. First, ankle extension moments during the back load lifting in humans were much higher than during leg load lifting (Figure 8), suggesting that perhaps central excitatory input from an extensor center to SO motoneurons was higher in former task, which could explain in part the higher mean SO EMG during back load lifting as opposed to leg lifting (Figure 12D). We also found no significant increase in mean SO EMG activity from 5- to 15%-back lifting even though the latter task required a greater ankle extension moment and MG EMG activity (Figure 8). Assuming there would be increased input to all ankle extensor motoneurons with 15% load vs. 5% load, this may suggest a net cancelation effect on SO due to additional inhibitory inputs.

There may be at least two neural mechanisms responsible for potential inhibition of slow-twitch motor units during fast tasks that require high power output. Eccles and Friedman with their colleagues (Eccles et al., 1961; Friedman et al., 1981) have found evidence in experiments on cats that the average size of recurrent inhibition via Renshaw cells measured at resting motoneuron membrane potential was greater in slow-twitch motor units than in fast-twitch ones, and suggested that this mechanism could contribute to selective inhibition of slow-twitch motor units during fast movements. However, when recurrent inhibitory post-synaptic potentials were measured at the membrane potential corresponding to the motoneuron firing threshold, no difference in recurrent inhibition between slow and fast motor unit types could be detected (Hultborn et al., 1988a,b; Lindsay and Binder, 1991). Thus, recurrent inhibition via Renshaw cells does not seem a probable mechanism for potential inhibition of SO motor units.

Another possible mechanism responsible for potential inhibition of SO motoneurons could be the force-dependent inhibition of SO from MG mediated by the Golgi tendon organ afferents (Nichols, 1994, 1999). This mechanism is consistent with the idea that in order to increase the mechanically advantageous contribution of MG to the combination of ankle extension and knee flexion moments, the contribution of one-joint SO to the ankle extension moment should be reduced (Prilutsky, 2000a).

## Author contributions

Ricky Mehta and Boris I. Prilutsky conceived the study, conducted surgeries and experiments, analyzed the data, wrote the manuscript and approved its last version.

### Conflict of interest statement

The authors declare that the research was conducted in the absence of any commercial or financial relationships that could be construed as a potential conflict of interest.
